# Effect of intramedullary nail and locking plate in the treatment of proximal humerus fracture: an update systematic review and meta-analysis

**DOI:** 10.1186/s13018-019-1345-0

**Published:** 2019-08-30

**Authors:** Xiaoqing Shi, Hao Liu, Runlin Xing, Wei Mei, Li Zhang, Liang Ding, Zhengquan Huang, Peimin Wang

**Affiliations:** Department of Orthopedic Trauma, Affiliated Hospital of Nanjing University of Chinese Medicine, Jiangsu Provincial Hospital of Traditional Chinese Medicine, 155 Hanzhong Road, PO Box 210029, Nanjing, China

**Keywords:** Proximal humeral fracture, Intramedullary nail, Locking plate, Internal fixation, Meta-analysis

## Abstract

**Background:**

To evaluate the effect of intramedullary nail and locking plate in the treatment of proximal humerus fracture (PHF).

**Methods:**

China National Knowledge Infrastructure (CNKI), Chinese Scientific Journals Database (VIP), Wan-fang database, Chinese Biomedicine Database (CBM), PubMed, EMBASE, Web of Science, and Cochrane Library were searched until July 2018. The eligible references all show that the control group uses locking plates to treat PHF, while the experimental group uses intramedullary nails to do that. Two reviewers independently retrieved and extracted the data. Reviewer Manager 5.3 was used for statistical analysis.

**Results:**

Thirty-eight retrospective studies were referred in this study which involves 2699 patients. Meta-analysis results show that the intramedullary nails in the treatment of proximal humeral fractures are superior to locking plates in terms of intraoperative blood loss, operative time, fracture healing time, postoperative complications, and postoperative infection. But there is no significance in constant, neck angle, VAS, external rotation, antexion, intorsion pronation, abduction, NEER, osteonecrosis, additional surgery, impingement syndrome, delayed union, screw penetration, and screw back-out.

**Conclusions:**

The intramedullary nail is superior to locking plate in reducing the total complication, intraoperative blood loss, operative time, postoperative fracture healing time and postoperative humeral head necrosis rate of PHF. Due to the limitations in this meta-analysis, more large-scale, multicenter, and rigorous designed RCTs should be conducted to confirm our findings.

**Trial registration:**

PROSPERO CRD42019120508

## Background

PHF is the third common limb fracture, accounting for 4 to 5% of total body fractures [1]. The incidence is located after hip fracture and distal radius fracture [2]. Most proximal humeral fractures occur in the elderly population. With the gradual arrival of the elderly society, the incidence has increased nearly threefold in the past 30 years [3–5]. There is no uniform standard for the diagnosis and treatment of proximal humeral fractures. Different treatment methods have their own advantages and disadvantages [6]. Most of the simple humeral greater tuberosity fractures are not obvious and can be treated conservatively, but there is still a risk of secondary displacement during conservative treatment [7, 8]. For patients with significant shifts, surgical treatment is recommended. Plate internal fixation is a more common method, which provides a reliable internal fixation for patients with second, third, and fourth fractures, but it has great damage to tissues and blood vessels [9–11]. The intramedullary nail has less soft tissue and less damage to the periosteum and blood vessels and can achieve minimally invasive effects [12]. Intramedullary nails are mainly used for the treatment of fractures of the second and third parts of the proximal humerus. A series of reports of intramedullary nails have achieved satisfactory results in the treatment of proximal humeral fractures [13, 14].

Due to the good biomechanical properties of the locking plate and the intramedullary nail, its exact clinical efficacy has become the main treatment [15–19]. However, due to the difference in the principle of internal fixation biomechanics and the surgical method, its efficacy and application are still unclear in clinical practice. According to our understanding, in recent years, relevant scholars have conducted several meta-analyses, but the analysis of postoperative indicators is incomplete, especially the analysis of postoperative complications. In the past 5 years, the comparative analysis of intramedullary nails and locking plates in the treatment of proximal humeral fractures has gradually increased, and we have included more studies. Finally, we conducted a meta-analysis of 38 studies.

## Methods

### Database and searching strategies

A literature retrieve was carried out in eight databases from their inception to July 2018, like CNKI, VIP, Wan-fang database, CBM, PubMed, EMBASE, Web of Science, and Cochrane Library. Search terms including “Proximal humerus fracture,” “Intramedullary nail,” “Locking plate,” and “Internal fixation” were used individually or in combination. The publishing language was restricted to Chinese and English.

### Inclusion criteria

The inclusion criteria are as follows: (i) internal fixation of displaced proximal humeral fractures; (ii) included both locking plates and intramedullary nails; (iii) greater than a minimum of 6 months of follow-up; (iv) a minimum of 21 patients for a given study; and (v) clinical outcomes during follow-ups included at least one of the following: intraoperative blood loss, operative time, fracture healing time, postoperative complications and postoperative infection, constant, neck angle, VAS, external rotation, antexion, intorsion pronation, abduction, NEER, osteonecrosis, additional surgery, impingement syndrome, delayed union, screw penetration, and screw back-out.

### Exclusion criteria

The exclusion criteria are as follows: (i) non-humeral proximal fracture; (ii) treatment mode non-locking plate or intramedullary nail treatment; (iii) non-clinical researches, basic researches, and review articles were excluded, as case reports and theoretical discussions; (iv) improper statistical methods, data defect literature; (v) genetic research; (vi) grey literature; and (vii) letters to editor.

### Data extraction

Two investigators (Xiaoqing Shi and Hao Liu) independently extracted and screened the data according to the inclusion criteria. We extracted the general details, such as patients’ characteristics, interventions, and outcomes, and a cross-check was done. Any disagreements were resolved through discussion or verification by a third investigator (Runlin Xing).

### Quality assessment

The quality of the non-randomized controlled trials was assessed by the MINORS entry, and trials with MINORS scores > 12 were included in the study [20]. The methodological quality and risk of bias of RCTs used the Cochrane Handbook.

### Statistical analysis

Revman 5.3 software was employed to pool the effect size. Mean difference (MD) or standardized mean difference (SMD) and 95% confidence intervals (CIs) were used for continuous variables. For the two-category data, we used OR (odds ratio)/RR (risk ratio) and 95% CIs as the efficacy analysis statistic. Heterogeneity was evaluated statistically using the *χ*^2^ test and inconsistency index statistic (*I*^2^). If substantial heterogeneity existed (*I*^2^ > 50% or *P* < 0.05), a random effect model was applied; otherwise, we adopted a fixed effect model [21]. Sensitivity analyses were explored to ensure the potential sources of heterogeneity and inspect the stability of the result. Evaluation of publication bias was made by plotting the funnel plot.

## Results

### Search results

**Fig. 1:**
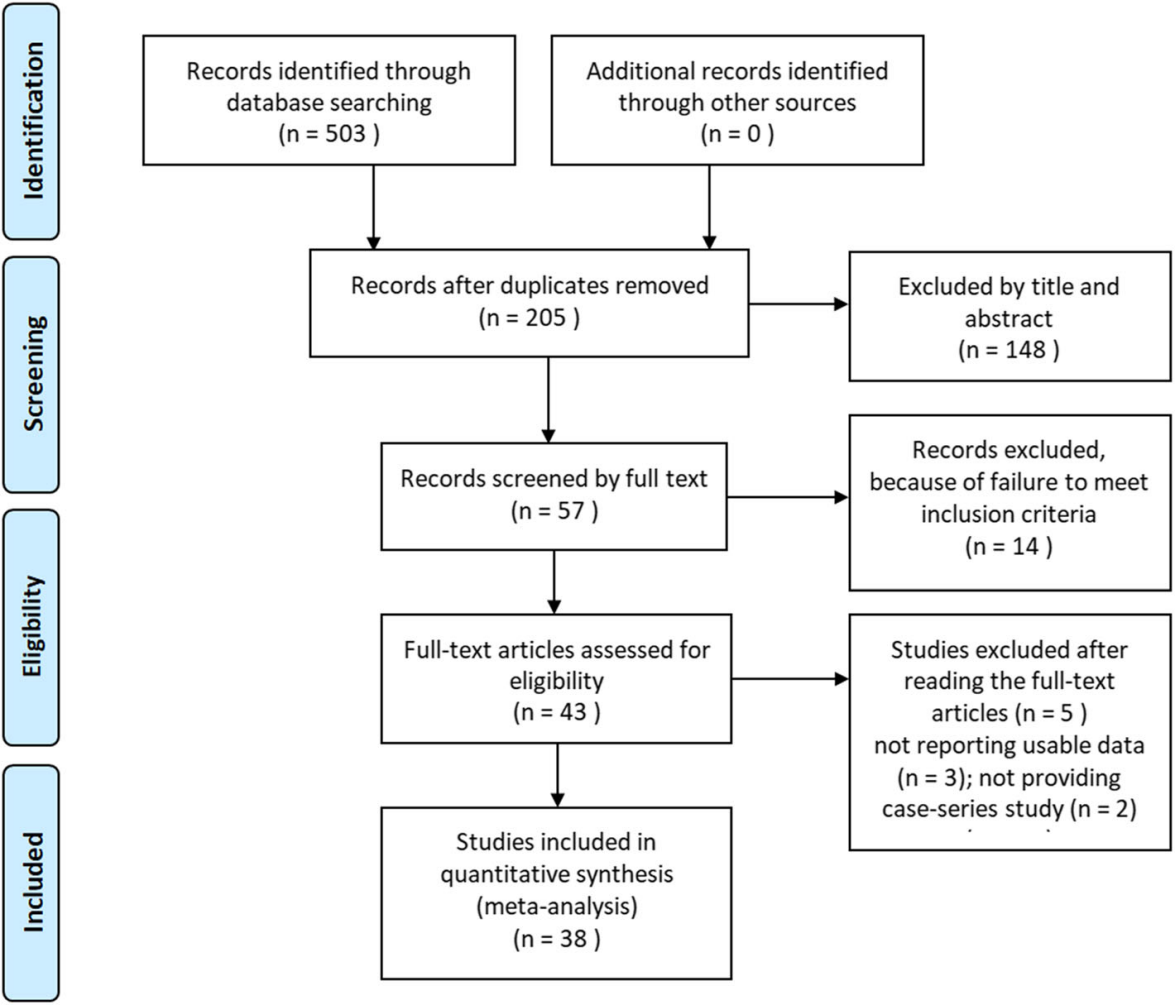
Flow chart of studies selecting

A total of 506 articles were initially obtained through the search strategy. After excluding 298 duplications, the remaining articles were screened based on their titles and abstracts, and 148 records were removed. By reading the full text, 14 literatures that did not meet the inclusion criteria were excluded. Finally, 38 trials [22–59] were enrolled in the systematic review and meta-analysis. The flowchart of the process for literature retrieval was shown in Fig. 1.

### Study characteristics

There were a total of 2699 patients (1238 in the locking plate group and 1461 in the intramedullary nail group) enrolled in our studies. More details of the included studies were presented in Table 1.

**Table 1:** Characteristics of the 38 studies included in the meta-analysis

Author	Age		Test type	Case	NEER classification
	(E) (years)	(C) (years)		(E/C)	(IN/LP)
Bi et al. [22]	67.2 + 4.9	65.5 + 6.8	Retrospective study	26/34	II, 60
Boudard et al. [23]	64.1 + 15.8	49.6 + 17.5	Retrospective study	30/33	III, 21; IV, 9/III, 21; IV, 12
Chen and Chen [24]	61.86 + 5.03	62.03 + 5.14	Retrospective study	46/64	II/III
Cheng et al. [25]	54.0 + 12.5	55.8 + 13.3	Retrospective study	54/54	III, 32; IV, 22/III, 36; IV, 18
Cui et al. [26]	55.4 + 5.8	57.5 + 5.8	Retrospective study	23/25	II, 48
Ding et al. [27]	74.3 + 3.4	74.2 + 3.3	Retrospective study	25/60	II, 17; III, 8/II, 37; III, 23
Dong et al. [28]	4.02 + 0.78	3.92 + 0.88	Retrospective study	17/32	II, 11; III, 6/II, 21; III, 10; IV, 1
Gadea et al. [29]	64	57	Retrospective study	54/53	IV, 107
Gracitelli et al. [30]	64.5 + 9.3	66.4 + 8.1	RCT	32/33	II, 16; III, 16/II, 16; III, 17
Gradl et al. [31]	63 + 16	63 + 16	Prospective	76/76	II, 52; III, 60; IV, 40
Ke [32]	51.3 + 4.6	50.2 + 4.9	Retrospective study	40/40	II, 80
Konrad et al. [33]	64.8 + 13.0	65.4 + 15.6	Prospective	58/153	III, 211
Li [34]	74	76	Retrospective study	29/25	II, 12; III, 17/II, 11; III, 14
Li et al. [35]	74	76	Retrospective study	29/25	II, 12; III, 17/II, 11; III, 14
Matziolis et al. [36]	55.6 + 16.5	54.8 + 16.9	Retrospective study	11/11	II, 22
Pan et al. [37]	69.2 + 8.83	69.15 + 8.08	Retrospective study	30/40	III, 19; IV 14/III, 23; IV, 17
Pu [38]	55.8 + 4.7	56.6 + 4.3	Retrospective study	27/27	NA
Qi [39]	NA	NA	Retrospective study	31/38	II/III
Shao et al. [40]	55.9 + 12.4	55.7 + 12.3	Retrospective study	34/34	II, 30; III, 21
Shen [41]	67	67	Retrospective study	22/24	II, 46
Shi et al. [42]	65.4 + 4.5	61.7 + 5.9	Retrospective study	33/37	II, 17; III, 21/II, 21; III, 16
Sui et al. [43]	59.61 + 6.71	58.32 + 6.54	Retrospective study	15/15	II, 4; III, 7; IV, 4/II, 5; III, 7; IV, 3
Tian et al. [44]	56.3 + 4.6	56.3 + 4.6	Retrospective study	30/30	NA
Trepat et al. [45]	64.5	68.3	Prospective	15/14	II, 29
Urda et al. [46]	70.92 + 11.4	71 + 13.54	Retrospective study	26/15	II, 41
Wang et al. [47]	63.4	63.4	Retrospective study	48/55	II, 21; III, 19; IV, 8/II, 3; III, 14; IV, 38
Wang [48]	61.8 + 4.7	62.2 + 4.1	Retrospective study	28/40	II, 68
Wang and Sheng [49]	76.9 + 4.5	76.5 + 4.7	Retrospective study	45/45	NA
Wu [50]	47.5 + 2.5	48.8 + 1.5	Retrospective study	43/43	NA
Xu et al. [51]	56.0 + 17.4	64.6 + 16.2	Retrospective study	14/24	II, 8; III, 5; IV, 1/II, 7; III, 11; IV, 6
Xue [52]	65.15 + 8.74	66.14 + 8.81	Retrospective study	40/40	II, 27; III, 13/II, 26; III, 14
Yu [53]	62.7 + 10.5	61.9 + 11.2	Retrospective study	46/46	II, 19; III, 22; IV, 5/II, 18; III, 19; IV, 9
Yu et al. [54]	59.3	59.3	Retrospective study	26/26	II, 20; III, 5; IV, 1/II, 21; III, 4; IV, 1
Zhou et al. [55]	46.46 + 5.78	43.45 + 6.34	Retrospective study	25/26	II, 30; III, 21
Zhou et al. [56]	65.2 + 3.6	64.5 + 4.7	Retrospective study	63/64	III, 127
Zhu et al. [57]	54.8 + 17.1	50.5 + 19.9	RCT	25/26	II, 51
Lekic et al. [58]	60	59	Retrospective study	12/12	II, 24
Tamimi et al. [59]	65.3	65.3	Retrospective study	10/22	II, 8; III, IV, 2/II, 2; III, IV, 20

*M* male, *F* female, *E* experiment group, *C* control group, *NA* not mentioned, *IN* intramedullary nail, *LP* locking plate

### Intraoperative blood loss

**Fig. 2:**
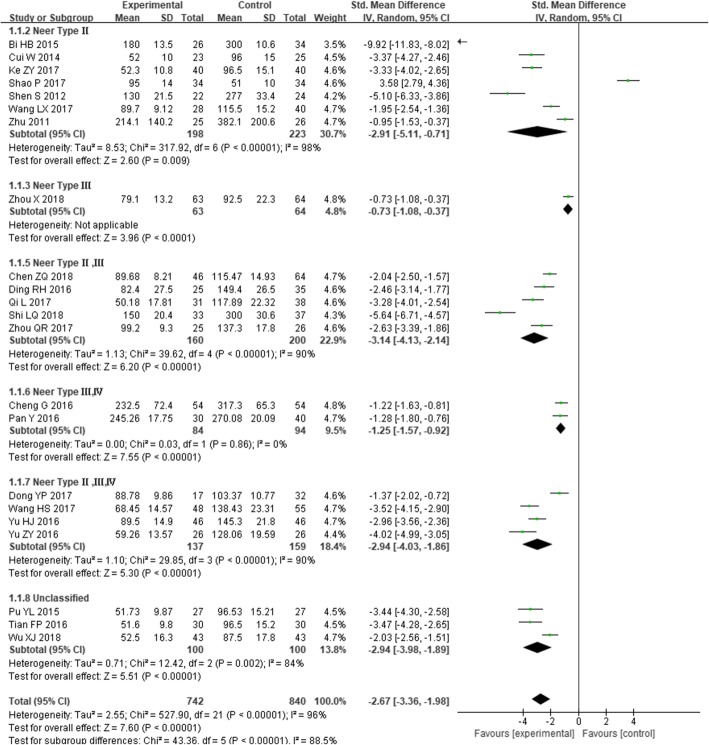
The forest plot for Intraoperative blood

Twenty-two studies [22, 24–28, 32, 37–42, 44, 47, 48, 50, 53–57] reported intraoperative blood loss, including 742 cases in the experimental group and 840 cases in the control group, *I*^*2*^ = 96%, *P* < 0.00001, and the heterogeneity was high. Therefore, the random effect model was used to calculate the combined effect. The results showed that intramedullary nail in the treatment of PHF is statistically significant, as its intraoperative blood loss is less than the locking plate [SMD = − 2.67, 95% CI (− 3.36, − 1.98), Fig. 2].

### Operation time

**Fig. 3:**
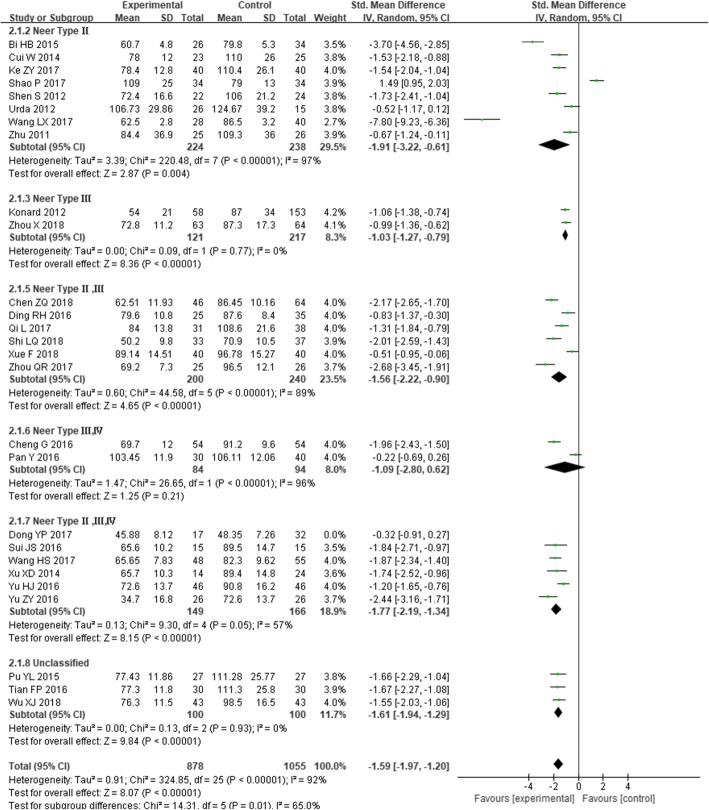
The forest plot for operation time

A total of 878 cases in the experimental group and 1055 cases in the control group, in 26 studies [22, 24–28, 32, 37–44, 46–48, 50–55, 57], had reported that the operation time, including 878cases in the experimental group and 1055 cases in the control group, *I*^*2*^ = 92%, *P* < 0.00001, and the heterogeneity was higher. Therefore, the random effect model was used to calculate the combined effect. The results showed that intramedullary nailing for the treatment of PHF was statistically significant in reducing surgical time compared with locking plates [SMD = − 1.59, 95% CI (− 1.97, − 1.20), Fig. 3].

### Fracture healing time

**Fig. 4:**
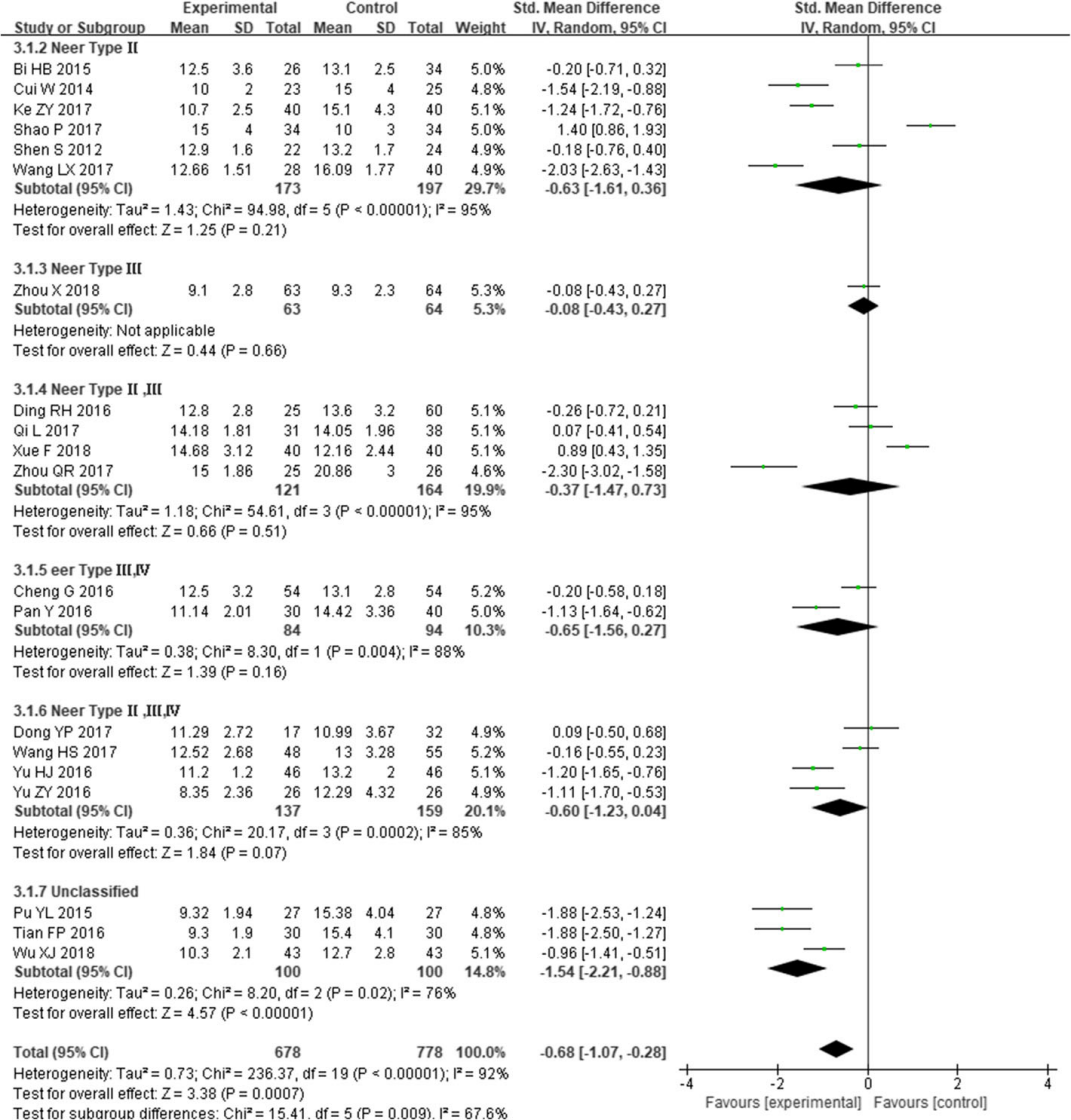
The forest plot for fracture healing time

Twenty studies [22, 25–28, 32, 37–41, 44, 47, 48, 50, 52–56] reported fracture healing time, including 678 cases in the experimental group and 778 cases in the control group, *I*^*2*^ = 92%, *P* < 0.00001, and the heterogeneity was high. Therefore, the random effect model was used to calculate the combined effect. The results showed that intramedullary nailing for the treatment of PHF was statistically significant in reducing surgical time compared with locking plates [SMD = − 0.68, 95% CI (− 1.07, − 0.28), Fig. 4].

### Overall complication

**Fig. 5:**
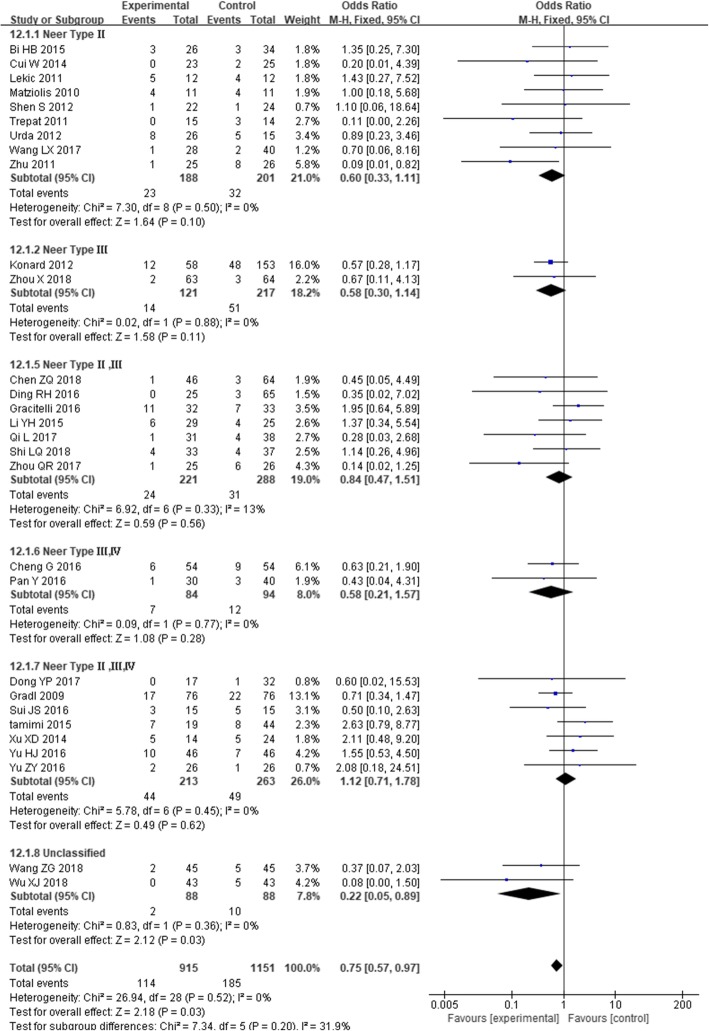
The forest plot for overall complication

Complications were reported in 29 studies [22, 24–28, 30, 31, 33, 35–37, 39, 41–43, 45, 46, 48–51, 53–59], including 915 cases in the experimental group and 1151 cases in the control group, *I*^*2*^ = 0%, *P* = 0.52, and there was no heterogeneity. Thus, the combined effect model was used to calculate the combined effect. The results showed that intramedullary nailing for the treatment of PHF was better than the locking plate in the incidence of complications [OR = 0.75, 95% CI (0.57, 0.97), Fig. 5].

### Other outcomes

We also analyzed other outcome indicators. Detailed information was shown in Table 2.

**Table 2:** Other outcome indicators

Postoperative index	Case	OR/SMD	*P*	*I*^2^ (%)
	(E/C)	(95% CI)		
Neck angle	323/325	0.02 (− 0.14, 0.18)	0.62	0
VAS	162/191	− 0.76 (− 1.91, 0.39)	< 0.00001	96
External rotation	294/315	0.01(− 0.29, 0.31)	0.0009	70
Antexion	266/277	− 0.09 (− 0.27, 0.08)	0.002	70
Intorsion pronation	137/169	− 0.01 (− 0.43, 0.41)	0.02	69
Abduction	137/169	− 0.11 (− 0.52, 0.30)	0.02	68
NEER	205/256	0.19 (− 0.14, 0.53)	0.01	68
Osteonecrosis	354/445	0.80 (0.37, 1.74)	0.76	0
Screw penetration	440/549	0.62 (0.35, 1.09)	0.57	0
Additional surgery	406/397	1.06 (0.69, 1.64)	0.32	13
Screw back-out	298/410	1.43 (0.67, 3.04)	0.31	14
Impingement syndrome	229/241	1.02 (0.51, 2.05)	0.63	0
Delayed union	474/721	0.74 (0.38, 1.44)	0.75	0
Constant	559/578	− 0.01 (− 0.13, 0.11)	0.51	0
Postoperative infection	341/490	0.37 (0.16, 0.85)	0.99	0

*E* experiment group, *C* control group, *OR* odds ratio, *SMD* standardized mean difference

### Quality assessment

For the methodological quality and risk of bias of RCTs, we used the Cochrane Handbook for Systematic Reviews of Interventions 5.2.0 for evaluation. The results showed that no studies used double blindness. On the other hand, for non-RCTs studies, we used MINORS to assess the methodological quality of the included studies. The results showed that the score interval was 13–18 points. Specifically, 13 points for five studies, 14 points for nine studies, 15 points for ten studies, 16 points for three studies, 17 points for seven studies, and 18 points for four studies. In general, this meta-analysis has qualitative limitations and most of the included studies had high risk of bias and low methodological quality.

### Sensitivity analysis and publication bias

**Fig. 6:**
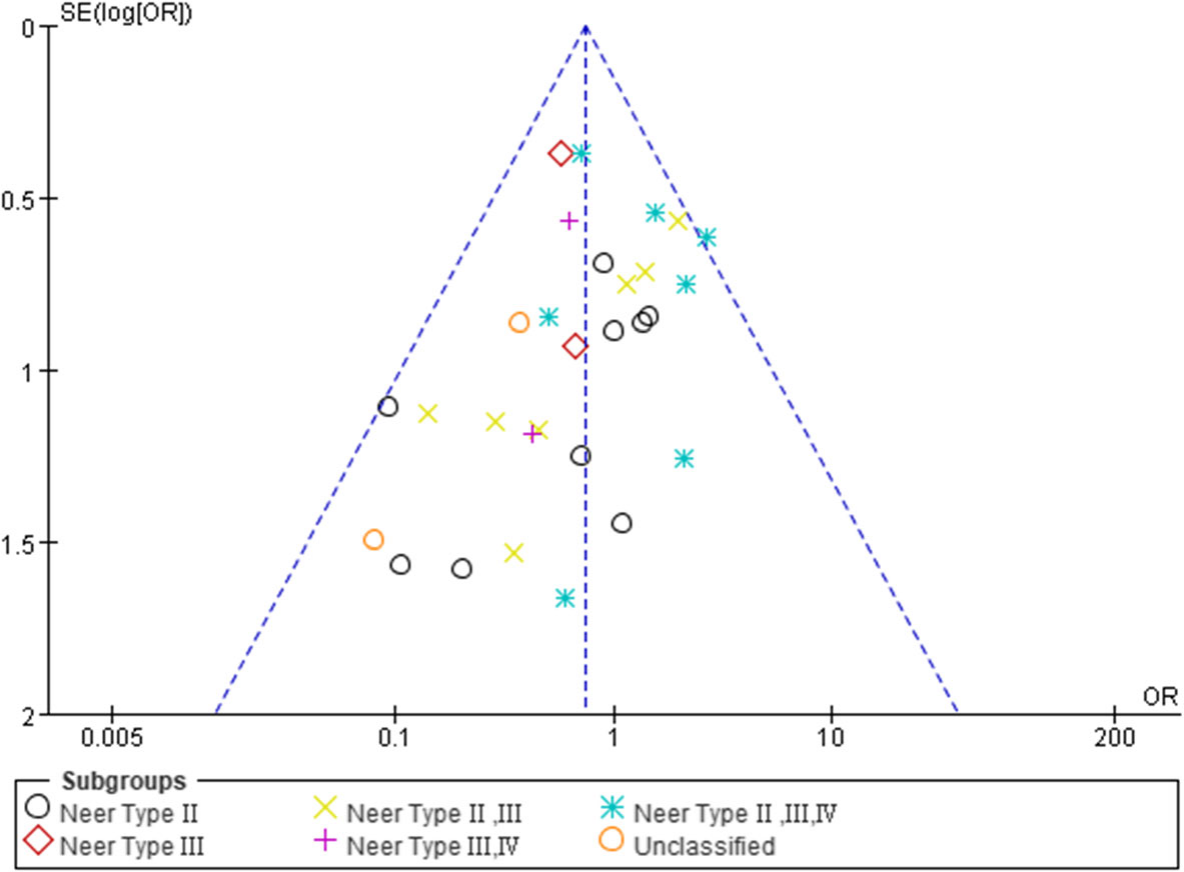
Funnel plot

To further confirm the stability of the above outcomes, we replaced the fixed effect model with random effect model and excluded the most and least weighted trials. Comparing with previous results, the outcome exhibited no obvious difference which revealed that our study was robust and reliable. We mainly assessed the publication bias of overall complications (Fig. 6). The results manifested there was no obvious publication bias in our analysis. However, most of the included studies were published in mainland China, and potential publication bias still likely existed.

## Discussion

In recent years, intramedullary nail and locking plate have been the main choices of internal fixation for PHF. From the biomechanics analysis, Edwards et al. [60] established an in vitro biomechanical comparison of an unstable humeral surgical neck fracture model and found that the locking plate has obvious advantages in bending resistance and torsion resistance. Relative to the eccentric fixation of the locking plate, Lekic [58] believed that the central fixation of the intramedullary nail can resist the greater varus force which is generated by the shoulder sleeve and the attached muscle. Biomechanics also shows that the axial load, torsional load, and bending load of the surgical neck fracture of the humerus are higher than that of the plate. Kitson [17] et al. also found that intramedullary nails have better stability in terms of eversion, flexion, and extension. Foruria [61] pointed out that there is no difference in dynamic torsional and static torsion resistance when treating proximal humeral fractures. Also both fixations provide stable biomechanical fixation, but the locking plate has better static torsional resistance. The previous researcher has conducted evidence-based medical analysis [62–66]; however, the conclusions reached are more limited, as the limited literature included and the types of research are mixed. In the past 3 years, many clinical experts have done more discussions on this. Based on the published literatures and incorporated new researches in recent years, we developed more stringent inclusion and exclusion criteria and reached a series of new conclusions.

The results of this meta-analysis show that (1) intramedullary nail in the treatment of PHF, intraoperative blood loss, operation time, fracture healing time, postoperative complications, and postoperative infection is better than locking plate treatment; (2) there were no significant differences in constant, neck angle, VAS, external rotation, antexion, intorsion pronation, abduction, NEER, osteonecrosis, additional surgery, impingement syndrome, delayed union, screw penetration, screw back-out between intramedullary nail, and locking plate in the treatment of proximal humeral fractures; (3) the screw back-out rate of the two-part fracture is better than the intramedullary nail in the locking plate; the shoulder anterior flexion angle intramedullary nail of the four-part fracture is better than the locking plate.

In terms of follow-up constant score, the intramedullary nail was not superior to the locking plate, and the results were not statistically different. Some studies concluded that may be related to surgical techniques [67–71]. In the meta-analysis done by Wang et al. [62], the same conclusions were obtained in terms of postoperative Constant score. von Ruden [72] also pointed out that both intramedullary nails and locking plates are suitable methods for the treatment of proximal humeral fractures. And these internal fixations have no significant differences in clinical function and imaging findings. The cause of this outcome may be related to postoperative pain, functional activity, muscle strength, and shoulder mobility, as these markers constitute the constant score. In this article, we know that shoulder mobility and VAS are not statistically significant in two different surgical procedures (Table 2). However, since this article does not comprehensively evaluate the outcomes, its conclusions may be changed.

Previous studies have suggested that there is no difference in the time of fracture healing between the two internal fixations. Jiang pointed out that this bias may be related to that the research is not enough in this area [64]. In our study, more stringent inclusion and exclusion criteria were developed, and more recent literatures were included, and different conclusions were drawn. We presume the reason for this result is that intramedullary nail treatment has less effect on blood flow around the fracture and surrounding soft tissue and can provide relatively stable fixation strength.

In terms of the incidence of screw penetration, Konrad et al. [33] found that the intramedullary nail was lower than the steel plate and that the steel plate was considered to be eccentrically fixed, which was prone to screw cutting. However, our study found that the use of locking plate and intramedullary nail in the treatment of proximal humeral fractures was not statistically significant. The cause of this result may be the crushing of the medial column, the complex degree of fracture, and the different screw positions.

There was a statistically significant difference in the overall risk of postoperative complications between the two groups, which is different from previous evidence-based studies [13–17]. In our meta-analysis, the total complication rates were 16.1% and 12.5% for locking plates and the intramedullary nails, respectively. Among them, intramedullary nail therapy is superior to locking plate therapy in reducing the postoperative infection rate. However, in other postoperative complications, we did not find the difference between the two procedures. Among these outcomes, intramedullary nail treatment is more dominant, but not statistically significant. The possible reasons for this result are (1) the size of the incision in the intramedullary nail and the area of the incision exposed to air are relatively small, which is less likely to be infected than the long incision of the locking plate; (2) the operation time of the locking plate is longer than that of the intramedullary nail. But with more high-quality RCTs, the conclusions may be different, and we should be cautious about this conclusion.

The limitations of this study are as follows: (1) this study cannot examine the use of surgical instruments by various subjects and evaluate the skill level and proficiency of the surgeon, which may cause clinical heterogeneity and affect the reliability of the meta-analytical strength and conclusion; (2) the type of study is retrospective analysis, and there is risk of selective bias, which may affect the authenticity and reliability of the research results; (3) the lack of clinical randomized controlled study, and the level of evidence is not high; (4) the doctor’s procedure is not completely unified, bringing a part of clinical heterogeneity; and (5) in all trials, the manufacturers of intramedullary nails and locking plates are different, and their quality is not the same.

Clinical outcomes have the potential to improve with time because the rate of the postoperative index can change with time. Despite the shortcomings in this study, we still try to avoid the risk of bias during the analysis and try subgroup analysis. Sensitivity analysis showed that the study has good stability and clinical reference value.

Additionally, there are fewer reliable randomized controlled trials included in this article. The level of evidence was reduced. It is difficult to control bias or confounding factors effectively. The evaluation efficiency may be reduced, and there may be publication bias, selection bias, implementation bias, and measurement bias. The inverted funnel plot shows that the included literature is basically within 95% CI. The article has certain reference value, but its results and applications should be treated with cautious attitude. If there are more clinical randomized controlled trials in this area, then a reliable conclusion will be drawn.

## Conclusion

Intramedullary nailing for the treatment of proximal humeral fractures, intraoperative blood loss, operation time, fracture healing time, overall complication, and postoperative infection is better than locking plate treatment. In the treatment of proximal humeral fractures, the intramedullary nail and the locking plate are both mature. Before the proficiency of the technique, considering the treatment of proximal humeral fracture with intramedullary nail can bring effective results, such as reducing the surgical trauma, protecting the blood supply of the fracture end, promoting fracture healing, and reducing the occurrence of postoperative complications, especially the occurrence of postoperative infection. The author believes that intramedullary nail treatment is a better choice in the strict control of surgical indications. However, because the quality of the literature included in this study is various, there is a risk of bias. This conclusion needs to be demonstrated by more well-designed, high-quality, large-sample, multi-center, randomized, double-blind controlled clinical trials. In addition, the study and discussion of increasing related complications are conducive to obtaining more rigorous and objective clinical evidence.

## Data Availability

All data generated or analyzed during this study are included in this published article.

## Abbreviations

CBM: Chinese Biomedicine Database
CIs: Confidence intervals
CNKI: China National Knowledge Infrastructure
*I*^2^: Inconsistency index statistic
MD: Mean difference
OR: Odds ratio
PHF: Proximal humerus fracture
RCT: Randomized controlled trial
SMD: Standardized mean difference
VAS: Visual analog score
VIP: Chinese Scientific Journals Database
